# Insight into the effects of moisture and layer build-up on the formation of lead soaps using micro-ATR-FTIR spectroscopic imaging of complex painted stratigraphies

**DOI:** 10.1007/s00216-020-03016-6

**Published:** 2020-11-09

**Authors:** Elena Possenti, Chiara Colombo, Marco Realini, Cai Li Song, Sergei G. Kazarian

**Affiliations:** 1 Istituto di Scienze del Patrimonio Culturale, Consiglio Nazionale delle Ricerche, ISPC-CNR, Via R. Cozzi 53, 20125 Milan, Italy; 2 Department of Chemical Engineering, Imperial College London, South Kensington Campus, London, SW7 2AZ UK

**Keywords:** Micro ATR-FTIR spectroscopic imaging, Paint layers, Metal soaps, Lead white, FT-IR spectroscopy

## Abstract

Metal soaps are formed in paint layers thorough the reaction of metal ions of pigments and fatty acids of organic binders. In this study, micro-ATR-FTIR spectroscopic imaging was used to analyse the formation of lead soaps in oil-based paint layers in relation to their exposure to moisture sources. The investigations were carried out on authentic samples of complex stratigraphies from cold painted terracotta statues (Sacred Mount, Varallo, UNESCO) and different IR-active lead white pigments, organic materials, and lead soaps were discriminated. The saponification of selected paint layers was correlated to the conservation history, the manufacturing technique, and the build-up of layers. The presence of hydrophilic layers within the stratigraphy and their role as a further water source are discussed. Furthermore, the modifications experienced by lead-based pigments from the core of an intact grain of pigment towards the newly formed decay phases were investigated via a novel approach based on shift of the peak for the corresponding spectral bands and their integrated absorbance in the ATR-FTIR spectra. Qualitative information on the spatial distribution from the chemical images was combined with quantitative information on the peak shift to evaluate the different manufacture (lead carbonate, basic lead carbonate) or the extent of decay undergone by the lead-based pigments as a function of their grain size, contiguous layers, and moisture source. Similar results, having a high impact on heritage science and analytical chemistry, allow developing *up-to-date* conservation strategies by connecting an advanced knowledge of the materials to the social and conservation history of artefacts.

**Supplementary Information:**

The online version of this article (10.1007/s00216-020-03016-6) contains supplementary material, which is available to authorized users.

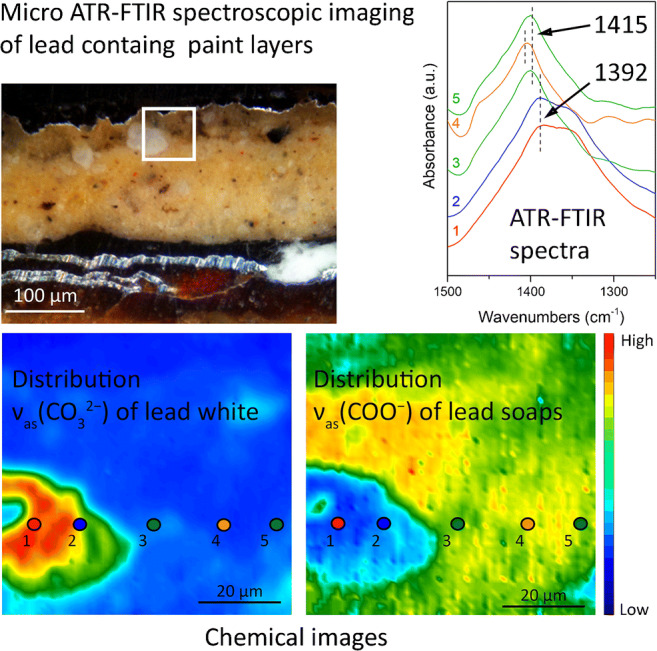

## Introduction

Metal soaps are formed within the painted layers of ancient preparations as a consequence of the interactions occurring between the metal ions of pigments and the fatty acids (especially free saturated fatty acids, SFAs) of organic binders [1]. The development, migration, and crystallization of such metal complexes are burning issues in conservation science [2], as the formation of these decay products (e.g. formation of spots, protrusions, craquelure, modification of colour and texture) has a direct influence on the preservation of the artefacts [3, 4].

Metal soaps are commonly detected in aged drying oils (even though they are also occasionally detected in proteic binders [5, 6] and terpenic media [7–10]); therefore, their characterization is an important part in the field of painting conservation. As a matter of fact, research in this topic is broad and constantly growing [2, 11, 12]. It is important to seek advanced knowledge in understanding the mechanisms governing the aging processes in order to develop the best conservation approaches targeting the formation of metal soaps [13–15]. In particular, great attention has been devoted to characterize the causes of the formation of metal soaps, including temperature [16, 17], pH [17], composition [16–18] and thickness of the paint layer [2], presence of water [16, 17], and the nature of the ground [4] and the conservation environment [4, 19].

Among these variables, humidity is a crucial factor for the formation of metal soaps in oil-based paint layers. In fact, it has been demonstrated that the exposure of drying oils to humidity, even in the short term, can have irreversible effects on the formation of metal soaps [17]. Furthermore, at the same humidity conditions, the development of different levels of decay phenomena (e.g. areas of the same painting showing heavy age craquelure alongside areas with minimal age craquelure) depends on the pigments (composition of the primary pigments and their reactivity when used in mixture to form new colours) and the organic materials which encompass a specific paint layer and the surrounding environment. These features have been extensively investigated on single-layer model samples [17] and on real-case stratigraphies [4, 19] having a relatively simple build-up of layers and composition.

In this study, the pigment-binder interactions in a highly complex scenario are considered, namely the stratigraphies of cold painted terracotta statues of the Sacred Mount of Varallo (Vercelli, north of Italy). These valuable artefacts, dating from the XVI–XVIII century and classified as precious items by the UNESCO, experienced several repainting and conservation treatments over the time for their important religious value. Nevertheless, the characterization and conservation of these pieces of art are very challenging.

These challenges include conservation under constant exposure to outdoor conditions, many paint layers with a huge number of different pigments in each paint layer (including the mixing of lead-based pigments with other pigments), as well as the presence of several organic materials, such as the coexistence of lipids, proteins, and terpenic resins, in micrometre-thin layers within the stratigraphy itself [20, 21]. It should be highlighted that the analytical approach to such complex systems is an analytical challenge itself. In fact, in the characterization of material from cultural heritage artefacts, many of these features are generally mutually present, they coexist down to the microscale, the samples (when available) are commonly of micrometre size and the investigation needs to obtain the maximum amount of information with the minimum alteration or damage for the sample.

The introduction of imaging techniques is a powerful advancement in analytical chemistry, as they allow to overcome the analytical limits of more conventional bulk techniques by identifying and localizing phases within a 2D or 3D region of interest, from the surface down to the core of a sample, in a non-destructive way [22]. Nowadays, the high potential of imaging approaches in the characterization of complex matrices and cultural heritage materials is indisputable, as demonstrated by recent studies on neutron imaging [23], diffraction tomography [24, 25], and spectroscopic imaging and mapping [26, 27].

Micro-ATR-FTIR (attenuated total reflection Fourier transform infrared) spectroscopic imaging is a powerful technique used to characterize organic and inorganic compounds with high chemical specificity and spatial resolution revealing the microstructure and chemistry of complex matrices. Its applications are well known in forensic science [28] and material, biological, and pharmaceutical science [29–31]. In the field of heritage science, micro-ATR-FTIR spectroscopic imaging pioneering studies have been carried out on albumen photographic prints [32] and paint stratigraphies [33]. In particular, Spring et al. have reported the potential of micro-ATR-FTIR spectroscopic imaging to investigate analytical issues frequently encountered in samples from paintings [33]. In their study, the increased sensitivity allowed the detection of trace materials and the spatial information derived about the location of compounds within the samples was particularly valuable, especially in the investigation of complex stratigraphies. Their approach demonstrated for the first time that it was possible to identify and localize lead soaps in chemical images collected from two paint samples from the National Gallery, London [33]. Therefore, the unique advantages of micro-ATR-FTIR spectroscopic imaging to obtain chemical images with high spatial resolution (down to ~ 3–4 μm in the fingerprint region) and without the need to microtome the paint samples were shown [33]. Nowadays, micro-ATR-FTIR spectroscopic imaging has been used to study metal carboxylates within different organic matrices including lipidic media [34–37] and terpenic varnishes [8, 9]. However, no micro-ATR-FTIR spectroscopic imaging data are available on the relationship between the exposure of paint layers to humidity and the formation-localization of lead soaps within the paint layers.

Here, the key feature of the study was to apply micro-ATR-FTIR spectroscopic imaging to complex stratigraphies of cold painted terracotta statues in order to investigate paint layers containing lead-based pigments and organic binders which have been constantly exposed to humidity. The artefacts are in chapels located in woodland, creating damp and relatively dark conditions. Furthermore, throughout the history of the terracotta statues, every paint layer experienced an initial phase of fresh application and exposure to the surrounding environment. This cycle occurred several times, as proved by the high number of paint layers forming the stratigraphies and by their interlayering with micrometre-thin layers of organic compounds and stucco layers. Moreover, it is worth highlighting that the lead-based layers have a remarkable thickness (tens of micrometres), which is an additional condition for the formation of lead soaps, and that all the layers were exposed to similar environmental conditions.

The following discussion aims to further investigate these features and to understand the causes by localizing the distribution of individual components in the paint layers (original materials and decay products) down to the microscale on authentic samples. The focus of the research was to characterize the spectroscopic features and spatial distribution of the IR-active lead-based pigments, organic media, and metal soaps in order to understand how the exposure to moisture has affected their formation and localization. Micro-ATR-FTIR spectroscopic imaging was also used to investigate paint layers permeable to moisture (also known as the hydrophilic materials, used not only as ground preparation but also within the stratigraphy) and to discuss their presence in the stratigraphies as an additional factor that influenced the formation of metal soaps.

The results allowed us to identify different lead white pigments within the stratigraphy and to localize them in different layers. The research also explored the compositional modifications experienced by lead white from the core of an intact grain of pigment towards the newly formed decay phases, all the way through the interface between the pigment and the binder. This was carried out via a novel approach based on shift of the peak for the corresponding spectral bands and their integrated absorbance in the ATR-FTIR spectra. Furthermore, we present in this study findings that can only be achieved by FTIR imaging and have a strong analytical impact for heritage science. In fact, by combining the qualitative information regarding the spatial distribution of components with the quantitative information from the peak shift in the spectra, it was possible to evaluate the decay undergone by the lead-based white pigments as a function of the humidity, the presence of the contiguous layers (organic and inorganic ones), and the grain size of lead white.

## Materials and methods

### Materials

This study was carried out on two micro-fragments sampled from two painted statues located in the V chapel of the Sacred Mount of Varallo (Vercelli, north of Italy; XVI–XVIII century; UNESCO World Heritage Site). The terracotta sculptures represent characters from the holy family, the history of Jesus, and the crucifixion. The statues were made of different elements of terracotta and the surface was painted with a peculiar cold painting technique. The main feature of these terracotta statues is that they experienced several repaintings and restorations over the centuries due to their frequent decay phenomena. It follows that the present stratigraphies can be considered a timeline of different “vocational iconographies” (which led to frequent changes in the colour of the surfaces depending on the historical period) and they are a record of the evolution of materials and painting techniques over the time, the interaction of new painting materials with the older decayed ones, and the influence of environmental conditions on their conservation.

Fig. S1 (see Electronic Supplementary Material, ESM) shows the statues from which individual fragments were sampled, the sampling regions, and the optical images of the stratigraphy of the two samples. The samples were collected during a diagnostic campaign carried out to support a conservation plan: one sample was taken from the right arm of the soldier, the other is the decoration of the tunic of the wise king. The samples, referred to as sample A and sample B respectively, were selected for this advanced micro-ATR-FTIR spectroscopic imaging study due to their intrinsic complexity. A scheme of the layer succession, as obtained from a preliminary investigation by conventional Raman spectroscopy and SEM-EDS, is reported in Table S1 in ESM.

Polished cross sections were prepared from paint micro-fragments by embedding them in slow-curing epoxy resin (Struers). After 24 h, the samples were cross-sectioned by using a microtome and submitted to dry polishing with conventional methods (silicon carbide papers with grit from 800 to 2500). The final wet polishing was carried out using in sequence 6.0 μm and 1.0 μm diamond polishing paste on polishing cloths.

### Methods

The identification of organic binders, their interaction with lead-based IR-active pigments, and the formation of metal soaps were investigated by micro-ATR-FTIR spectroscopic imaging.

Optical images were collected by a Leitz Ortholux microscope coupled with a Nikon DS-5M/USB camera and the Lucia Image software.

The micro-ATR-FTIR spectroscopic imaging measurements were carried out using a Agilent 670 FTIR spectrometer operating in continuous scan mode coupled to a Cary 600 IR microscope (Agilent, Inc.) equipped with a 64 × 64-pixel 2D focal plane array (FPA) detector (Santa Barbara, CA, USA.). Each pixel can collect a spectrum, leading to the simultaneous collection of 4096 FTIR spectra in any one measurement. The micro-ATR-FTIR measurements were performed with a × 15 Cassegrain objective with a slide-on germanium (Ge) crystal as the internal reflective element. The ATR-FTIR spectra were collected in the mid-IR range of 4000–900 cm^−1^ with a spectral resolution of 8 cm^−1^ and 64 co-added scans. The chemical images of the selected analytes were obtained by plotting the integrated absorbance of their corresponding spectral bands as a function of all pixels of the array. The chemical images, representing the spatial distribution of the absorbance of the specific spectral band-of-interest, were presented in a colourmap spanning from low absorbance (blue) to high absorbance (red). The estimated size of the chemical images obtained with this setup is ~ 70 × 70 μm^2^.

For a measurement to be taken, the sample was placed on a motorized stage and the stage was raised to bring the sample into contact with the Ge crystal. Multiple measurements were taken on the same region of interest (ROI) by gradually raising the stage to make sure good contact was made to obtain high-quality spectra. Due to the intrinsic heterogeneous composition of paint layers, the micro-ATR-FTIR imaging measurements were carried out in different areas of the polished cross section in order to ascertain that the collected chemical images are representative of the sequence and chemical composition of paint layers. Particular attention was given to ensure the imaging area of investigation always covered from two up to five layers of interest, so that the detection of spectroscopic differences can be unambiguously attributed to an intrinsic feature of the layers and not to variations in instrumental setup or experimental conditions. Therefore, thanks to the high-quality data obtained in the micro-ATR-FTIR images and to the specific approach used to collect them, slight differences in the ATR-FTIR spectra of the same (or similar) phases are considered reliable and they can be used to reveal actual differences in the chemical composition of the paint layers.

Chemical images were extracted from the Resolution Pro software (Agilent, Inc.). ATR-FTIR spectra representative of selected compounds were obtained from the imaging datasets by extracting them from individual pixels from the images. Where applicable, the spectra presented for discussion were averaged from three datapoints in the same contiguous region, to obtain spectra of higher signal-to-noise ratio. Further processing of the data, including the systematic extraction of ATR-FTIR spectra from a small ROI from the chemical images, the calculus of the area of a band within a vibrational range of interest, and the exact spatial position of the maximum absorbance of the corresponding spectral peaks, was done in MATLAB R2019b software (MathWorks Inc., USA).

## Results and discussion

### Differentiation of lead-based white pigments using micro-ATR-FTIR imaging

**Fig. 1:**
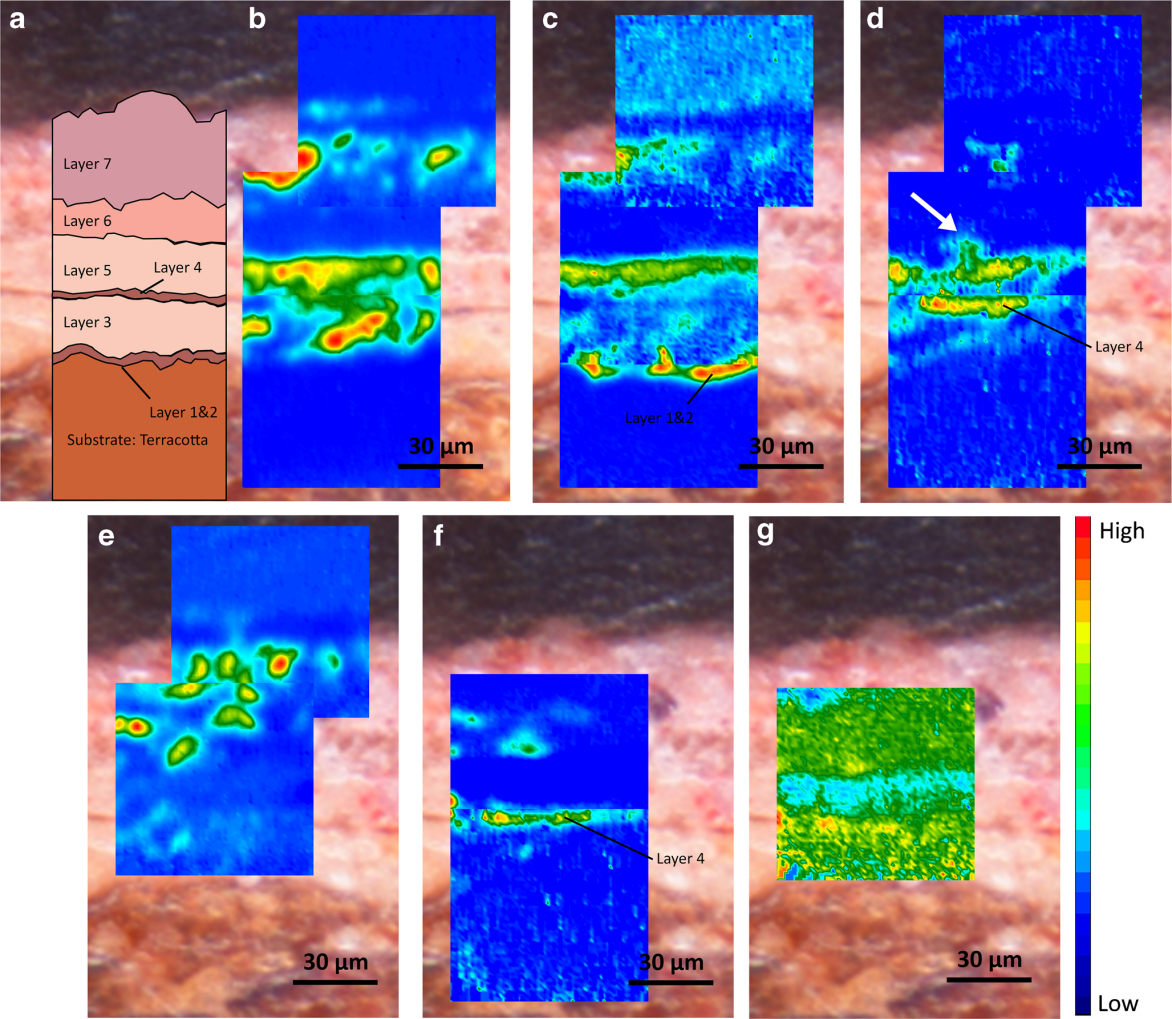
Sample A. (**a**) Optical microscopy image of the polished cross section showing the layer succession. Chemical images showing the distribution of (**b**) lead white (1482–1288 cm^−1^); (**c**) lipid—siccative oil (1780–1692 cm^−1^); (**d**) lead carboxylates (1560–1480 cm^−1^); (**e**) gypsum (1163–1056 cm^−1^); (**f**) protein (1667–1592 cm^−1^); and (**g**) calcium oxalates (1327–1310 cm^−1^). The chemical images are positioned in the exact region measured by micro-ATR-FTIR spectroscopic imaging. The white arrow in (**d**) indicates the protrusion formed by lead carboxylates

**Fig. 2:**
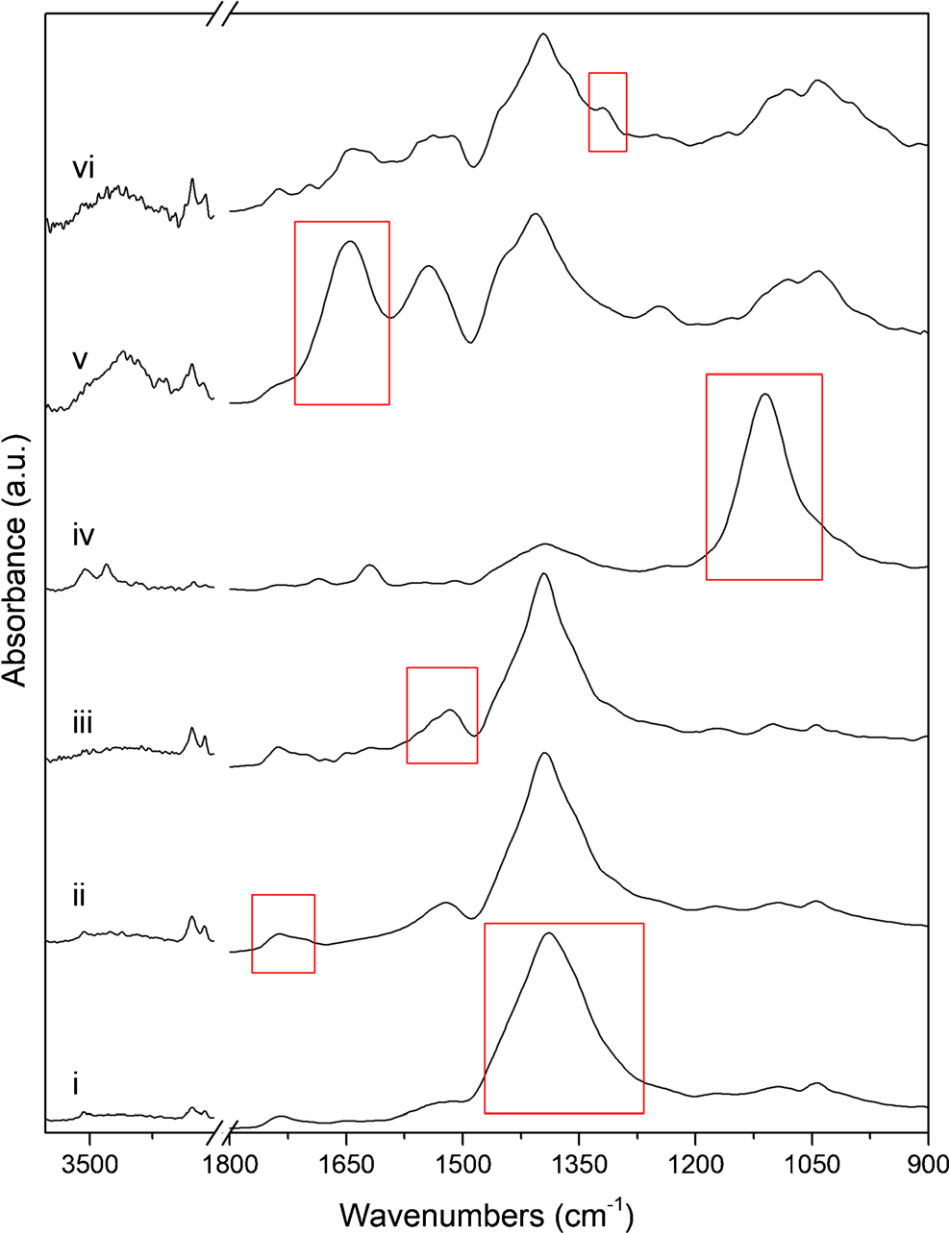
Micro-ATR-FTIR spectra of compounds detected in the chemical images of Fig. 3. The red rectangles indicate the integrated band for lead white (i), lipid—siccative oil (ii), lead soaps (iii), gypsum (iv), protein (v), and calcium oxalates (vi) used to generate the chemical images

Figure 1 shows a sequence of micro-ATR-FTIR images collected from sample A. The spectroscopic features of IR-active lead-based pigments were explored. The integrated spectral bands shown in Fig. 2 were used to generate the chemical images (Fig. 1) and the attribution of their vibrational modes is presented in Table 1. The description of the layer sequence can be found in Table S1 in ESM.

**Table 1:** Integrated wavenumber ranges, assignment of vibrational modes, and the peak wavenumber of the compounds detected in samples A and B. The anti-symmetric and symmetric stretching modes are denoted with *ν*_as_ and *ν*_sy_ respectively

Compounds	Integrated bands (cm^−1^)	Vibration mode	Peak wavenumber (cm^−1^)
Lead white—basic lead carbonate (synthetic analogue of hydrocerussite, 2PbCO_3_·Pb(OH)_2_)	1482–1288	*ν*_as_(CO_3_^2−^)	~ 1392–1384
Lead white—lead carbonate (synthetic analogue of cerussite, PbCO_3_)	1482–1288	*ν*_as_(CO_3_^2−^)	1365
Lead carboxylates	1558–1480	ν_as_(COO^−^)	1515
Lipid—siccative oil	1780–1692	*ν*_sy_(C=O ester)	1738
Gypsum	1163–1056	*ν*_as_(SO_4_^2−^)	1111
Protein	1667–1592	*ν*(C=O amide I)	1638
Calcium oxalates	1327–1310	*ν*_as_(CO)	1320

As clearly demonstrated by the chemical images, the stratigraphy is composed of a complex sequence of 7 different layers, including preparation layers (layers 1, 2, and 4) whose particular composition and function within the build-up of layers are out of the scope of the discussion here (ongoing paper is under way to discuss in depth the nature of these preparation layers). Instead, we focus on the results gathered from the composition of the four painted layers present in this sample. They can be distinguished into two different typologies: the first one, made of a mixture of gypsum with a very low fraction of protein and calcium oxalates (most likely formed by the decay and mineralization of the proteic binder of this layer; Fig. 1e–g), characterizes layer 6; the latter, based on an organic binder and lead-based pigments (detected by energy-dispersive X-ray spectroscopy in conjunction with scanning electron microscopy (SEM-EDS) investigations), is seen in layers 3, 5, and 7.

**Fig. 3:**
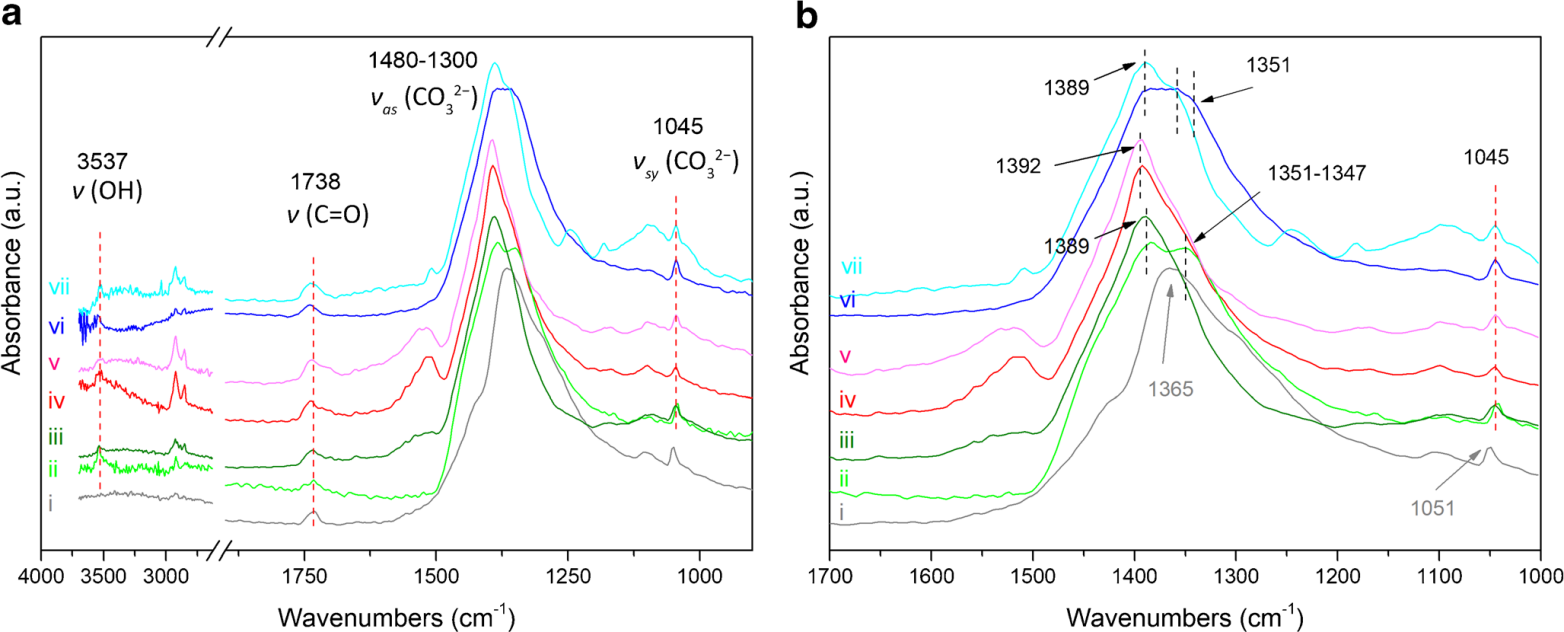
Sample A. (a) ATR-FTIR spectra of lead-based carbonate pigment as extracted from a single pixel of the chemical image of lead white. (b) The spectral region 1700–1000 cm^**−**1^. The ATR-FTIR spectra nos. i, ii, and iii are representative of layer 3, the ATR-FTIR spectra nos. iv and v are representative of layer 5, and the ATR-FTIR spectra nos. vi and vii are representative of layer 7. Annotation of the layer sequences can be found in Fig. 1a

Focusing on this last typology of layers, all of them are characterized by a lipidic binder (Fig. 1c), most probably ascribable to a siccative oil (such as linseed oil), as demonstrated by the vibrational bands located at 2924 cm^−1^, 2853 cm^−1^, and 1739 cm^−1^ assigned to the *ν*_as_(CH_2_), *ν*_sy_(CH_2_), and *ν*_sy_(C=O ester) of the triglyceride cross-linked network (Fig. 3 and Fig. S2 in ESM) respectively. The binder generally has a very low absorbance which suggests that, at this stage, the lipid is a residual component of the pink lead-based layers. The lipid residue is present in significantly lower amounts in comparison to the inorganic fraction. Focusing on the pigments, all of the ATR-FTIR spectra extracted corresponding to these pink layers show a very strong absorption band centred between 1480 and 1300 cm^−1^, owing to the asymmetric stretching vibration of the carbonate group (*ν*_as_(CO_3_^2−^)). In general, a further identification of the specific carbonate-based pigment is challenging by micro-ATR-FTIR imaging as the intrinsic cutoff of FPA detectors at about 920–900 cm^−1^ prevents the detection of important marker bands below this limit, such as the bending modes of carbonate group in the fingerprint region between 900 and 400 cm^−1^. However, in this case, the presence of a characteristic sharp peak at 1045 cm^−1^ of *ν*_sy_(CO_3_^2−^) is used to unambiguously identify this particular carbonate-based pigment (Fig. 3b) as basic lead carbonate [38, 39]. In addition, the detection of the characteristic absorption band at 3537 cm^−1^ attributed to the *ν*(OH) confirms the detection of the basic lead carbonate.

Interestingly, although the vibrational pattern of lead white is easily recognized in all the pink layers, there are some noticeable differences in their ATR-FTIR spectra. In particular, the lead white pigment of layer 3 and layer 7 shows the *ν*_as_(CO_3_^2−^) at 1389 cm^−1^, with a shoulder peak around 1351–1347 cm^−1^; meanwhile, the ATR-FTIR spectrum of lead white used in layer 5 has the *ν*_as_(CO_3_^2−^) band at 1392 cm^−1^ and a less pronounced shoulder peak at a lower wavenumber than that of layers 3 and 7 (Fig. 3c). On the other hand, no variations in the band position are observed for the peaks at 1045 cm^−1^ and 3537 cm^−1^.

Moreover, in the innermost lead-based pink layer (layer 3), it is possible to identify an ATR-FTIR spectrum characterized by a strong peak at 1365 cm^−^^1^ of *ν*_as_(CO_3_^2−^) and a relatively weaker peak at 1050 cm^−1^ of *ν*_sy_(CO_3_^2−^), as showed by the ATR-FTIR spectrum *i* of Fig. 3. These spectral bands are due to the presence of anhydrous lead carbonate (the synthetic analogue of cerussite, PbCO_3_). The two shoulders at 1427 cm^−1^ and 1296 cm^−1^, the weak peak at 1103 cm^−1^, and the absence of the OH stretching modes in the 3600–3500 cm^−1^ region as well as the agreeable match with the ATR-FTIR spectra from the RRUFF database (reference patterns: R040069, R050011, R050023, R050174, R050298, R060017; [40]) and comparison with literature data [39] unambiguously confirm the identification of cerussite in layer 3. On the basis of these results, lead white of layers 5 and 7 is composed only of basic lead carbonate (synthetic analogue of hydrocerussite) while lead white of layer 3 is composed by a mixture of basic lead carbonate and lead carbonate (synthetic analogue of cerussite). These findings are in line with literature data which have demonstrated that lead white may consists of basic lead carbonate, lead carbonate, or a mixture of both [41, 42]. Furthermore, the possibility to simultaneously study the distribution of lead carbonate and basic lead carbonate via micro-ATR-FTIR spectroscopic imaging may be a valuable complement to micro-XRD mapping of paint stratigraphies having complex mixtures of pigments with similar elemental composition [43]. In addition, the identification of anhydrous lead carbonate in layer 3 could be attributed to different lead white pigments employed in different historical periods [9, 11, 44, 45]. In fact, historical treatises describing the manufacturing procedures reveal that the final composition of lead white depends on the synthesis processes (stack processes in which sheets of lead were exposed to vinegar and/or manure) and on post-synthesis treatments (including washings, grinding of the pigment in water or in acidic media, heating in water). Considering that the chemical equilibrium during the pigment manufacture favours the precipitation of basic lead carbonate over lead carbonate under ambient conditions especially with high humidity, it is conceivable that lead white of layers 5 and 7 was synthesized under reaction conditions different from that of layer 3 [42, 46–48].

### Studies of the decay of lead white pigment and the formation of lead soaps

Lead soaps have been detected in lead-based layers of both the samples, and they are characterized by the strong absorption at 1515 cm^−1^ (ν_as_(COO^−^)) and a shoulder band at 1538 cm^−1^ [36, 44, 49, 50]. Nevertheless, further detailed discrimination of the specific lead carboxylates (palmitate, stearate, azelate) from the ATR-FTIR spectra is not feasible, as (1) the ν_as_(COO^−^) band is not as sharp as shown in literature [49, 51], probably due to a not so well-defined coordination geometry of the metal soaps (poorly crystalline metal soaps or metal soaps not fully crystallized); (2) the other marker bands of lead soaps (such as the progression of bands associated with the wagging and twisting of hydrocarbon long chains in the range between 1350 and 1150 cm^−1^ [16]) are of low absorbance and/or overlapped with the absorptions of other compounds; (3) possible co-presence of different lead soaps (starates, azelates, oleates); and (4) the possible formation of a lead ionomer both before or after the oligomerization of the drying oil, which partially contribute to the ν_as_(COO^−^) mode detected in the ATR-FTIR spectra [1]. In fact, lead soaps are always detected in mixtures with at least two other substances, typically oil of the binding medium (1738 cm^−1^, 2929 cm^−1^, and 2855 cm^−1^) and the lead white pigment (1045 cm^−1^). The co-presence of compounds (including the possible presence of a lead ionomer), existing even down to the microscale, results in the relatively low amount of lead soaps to be captured in the ATR-FTIR spectra, thereby making the identification unattainable [16].

The ATR-FTIR spectra of metal soaps of sample A are shown in Fig. S2 in ESM, while those of sample B will be discussed in the following.

The spatial distribution of lead soaps can be inferred from the chemical image of Fig. 1d. By comparing Fig. 1d and f, a graphical superimposition of the distribution of lead carboxylates and proteins can be observed in layer 4. However, this overlapping is only graphical, as there are no proteins in layer 5 and there are no carboxylates in layer 4. Focusing on lead soaps and the relative absorbance of their corresponding band, it is worth highlighting that lead soaps appear to be more abundant in layer 5, whereas they are less evident in layer 3 and layer 7. Interestingly, their relative abundance goes hand in hand with the absorbance of the lipid binder. More precisely, in regions where the absorbance of metal soaps is higher, the absorbance of oil bands is higher as well. This suggests that lead soaps are formed in a higher fraction in those regions where the oil-rich layer provides a major source of mobile and reactive fatty acids [2, 52]. This demonstrates that the availability and quantity of fatty acids play a crucial role in the formation of metal soaps. Moreover, the typical shape of lead soap protrusions is well distinguished in Fig. 1d by the rounded protruding shape at the interface between layer 5 and layer 6 (the gypsum-protein layer). This suggests that the relative abundance of carboxylic acids and free SFAs in layer 5 permits the migration and crystallization of metal soaps, more so closer to the outer surface of the paint layer, with the consequent formation of lead soap aggregates in bigger nuclei. Even though a migration of metal soaps towards the surface occurred, they did not form a compact crust over the paint layer and no remineralization of insoluble lead sulphates and oxalates was detected [1, 2, 52].

The more pronounced formation of lead carboxylates in layer 5 can be attributed to the possible original presence of a higher amount of oil, or to the composition of the oil itself, probably originally richer of SFAs. Nevertheless, recent research studies on the role played by the surrounding environment on the decay of oil-based media showed that water may play a crucial role in boosting the hydrolysis of the ester groups in oil and the descending formation of new reactive carboxylate groups. In fact, water would increase the reactivity of the binder in ancient preparation layers and the transformation of fatty acids in metal soaps [16, 17, 44].

In layers 3, 5, and 7, if the initial formation of metal soaps can be attributed to the intrinsic composition of the layers, starting from the drying processes and the formation of the ionomer network, an additional aspect needs to be considered. Taking into consideration that each one of the three lead-based layers has been progressively exposed to the atmospheric environment in a conservation context that is rich of moisture (the painted statues are located in a woodland setting and within chapels), something different has probably emphasized the formation of metal soaps in layer 5. This new factor can be identified as the water molecules coming from the surrounding layers. A possible source of water molecules could be the underlying layer (layer 4), composed of proteins, which constitutes a hydrophilic and hygroscopic substrate for layer 5. Furthermore, layer 5 could have been applied on an incompletely dried layer 4, i.e. a ground still rich in residual water.

That said, it is more likely that the principal source of moisture was from layer 6, a paint layer composed by a mix of gypsum and proteins (probably animal glue). This combination of materials is a rather common recipe used for grounds, reparation layers, or stuccoes and it is generally used to remodel lost portions of the statues. The mixture is generally applied in the form of a wet paste with proteins (swollen and dispersed in hot water) added to improve the mechanical properties of gypsum-based materials [53]. Following this reasoning, it can thus be hypothesized that due to the unavoidable aging process of the lead-based layer 5, the painter/restorer applied a new preparation layer in gypsum and animal glues to reshape the layer profile (layer 6) and to receive a new pink layer (layer 7). However, owing to the bad state of conservation of layer 5, part of the moisture used to prepare the gypsum layer penetrated into pores and micro-cracks of the paint layer providing the trigger for the development of a more advanced saponification process. Furthermore, it must be considered that the gypsum-protein layer remained hydrophilic even after curing; hence, its interactions with the surrounding environment (e.g. absorption of water vapour in air) affects the innermost decayed layers which, in fact, developed protrusions at their interface.

A further aspect deserves consideration. In this stratigraphy, minium (Pb_3_O_4_) and cinnabar (HgS) are detected by Raman spectroscopy in layer 5, that is to say, in the layer that experienced the most advanced decay having metal soaps and protrusions. Actually, the presence of minium in paint layers is not unusual, due to its cheap price as a pigment and optical properties [54]. Moreover, a mixture of minium with lead white and other red pigments (vermilion, red ochre) was typically employed to produce the flesh tones [55, 56], notwithstanding the well-known darkening experienced by lead-based pigments when mixed with sulphur-containing pigments (such as vermilion, cinnabar, HgS) [54, 57, 58].

However, it should be considered that lead white and lead oxides (in particular, minium) have different thermodynamic stability fields [11, 42, 59] and the exposure to the high humidity values of the basic lead carbonate is one of the main driving forces of its partial transformation in minium (in addition to lead soaps), which would be the most stable mineral phase. Thereby, considering that layer 5 does not show darkening notwithstanding its advanced decay, the presence of minium in this highly decayed paint layer could be more probably interpreted as a further decay product demonstrating the strong decay induced by water molecules (e.g. due to the atmospheric conditions of the chapel). In the complex decay of lead-based layers, the possible role played by past restorations is a further aspect to consider. In fact, the alteration of lead white on cold painted terracotta statues has been assumed as a consequence of aggressive conservation treatments with on acid or base products [60, 61] and past restorations are indirectly documented by the complex layer sequence in these samples. In addition, lead-based decay salts (as, e.g., cotunnite, PbCl_2_), whose formation is attributed to these kinds of conservation treatments [60, 61], have been already detected in paint samples originating from the same Sacred Mount of the painted statues here investigated [21].

The ATR-FTIR spectra of lead white and lead soaps show strong absorbances in the vibrational region 1410–1380 cm^−1^. As previously discussed, lead white has the *ν*_as_(CO_3_^2−^) centred at 1392 cm^−1^, whereas lead soaps have a typical ν_sy_(COO^−^) band at higher wavenumbers (1418 cm^−1^ and 1406 cm^−1^ for lead palmitates/stearates or for lead azelates, respectively) [49]. Here, the peak at 1410–1380 cm^−1^ is attributed only to lead white. This is because the spectral bands of lead soaps at 1515 cm^−1^ and between 1418 and 1406 cm^−1^ are of relatively weaker absorbance compared to that of lead white [49]; thus, the latter is convoluted/hindered in the very strong *ν*_as_(CO_3_^2−^) band at ~ 1410–1380 cm^−1^ of lead white [44, 50].

**Fig. 4:**
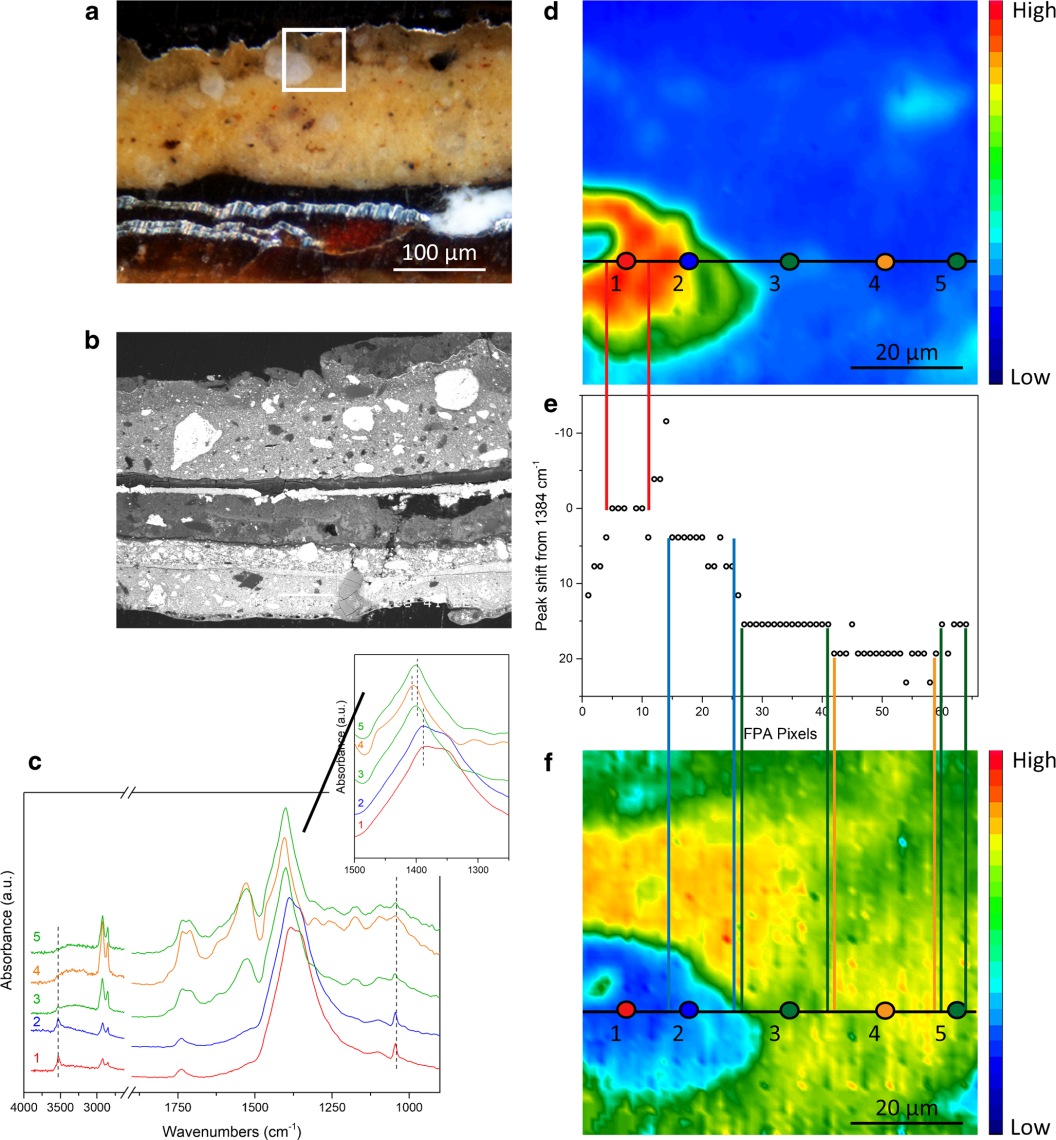
Sample B. (**a**) Optical microscopy image of the polished cross section showing the layer succession. The white square indicates the area investigated by micro-ATR-FTIR spectroscopic imaging (70 μm × 70 μm). (**b**) SEM backscattered electron image of the stratigraphy, showing the morphology of the most external layers and the co-presence of micrometric (~ 1–5 μm) and sub-micrometric lead white particles dispersed with bigger pigment grains (~ 50–100 μm). (**c**) Normalized ATR-FTIR spectra collected from the core of intact lead white (1), at the interface between the pigment and the binder (2), and within the binder in correspondence of lead soaps (3, 4, 5). The position from where the ATR-FTIR spectra were extracted is shown in images (**d**) and (**e**). (**d**) Chemical image showing the distribution of lead white pigment (integrated band: 1480–1200 cm^−1^). (**e**) Graph showing the peak shift of *ν*_as_(CO_3_^2−^) with respect to the reference peak of intact lead white. (**f**) Chemical image showing the distribution of lead carboxylates (integrated band: 1560–1480 cm^−1^). The two horizontal black lines in (**d**) and (**f**) indicate the ATR-FTIR spectra investigated to create graph (**e**). The vertical red lines show that in the corresponding area where lead white is clearly present, the peak shift is zero or positive, while the vertical blue, green, and orange lines show that in the regions where lead carboxylates are progressively more prominent, lead white is still present and its *ν*_as_(CO_3_^2−^) peak is significantly shifted to the higher wavenumber region.

The spectral position of the *ν*_as_(CO_3_^2−^) band allows further insight into the progressive evolution of intact lead white pigment to a decayed lead white phase, and the descending formation of lead soaps. A possible visualization of this phenomenon is illustrated by sample B in Fig. 4. The chemical images (Fig. 4d and f) show complementary spatial distribution of lead white and lead soaps. By exploring the ATR-FTIR spectra of representative regions where lead soaps are present, lead white vibrational bands are present as well (Fig. 4c). Additionally, the ATR-FTIR spectra reveal that the spectral position of the *ν*_as_(CO_3_^2−^) band is shifted depending on whether the ATR-FTIR spectra are obtained from the core of the intact lead white pigment, oil-and-lead-soaps regions, or the interface between the pigment and the oil binding media. The position of the *ν*_as_(CO_3_^2−^) band of lead white, measured in the core of intact pigment grains, is used as reference to study the extent of the peak shift of decayed lead white.

**Fig. 5:**
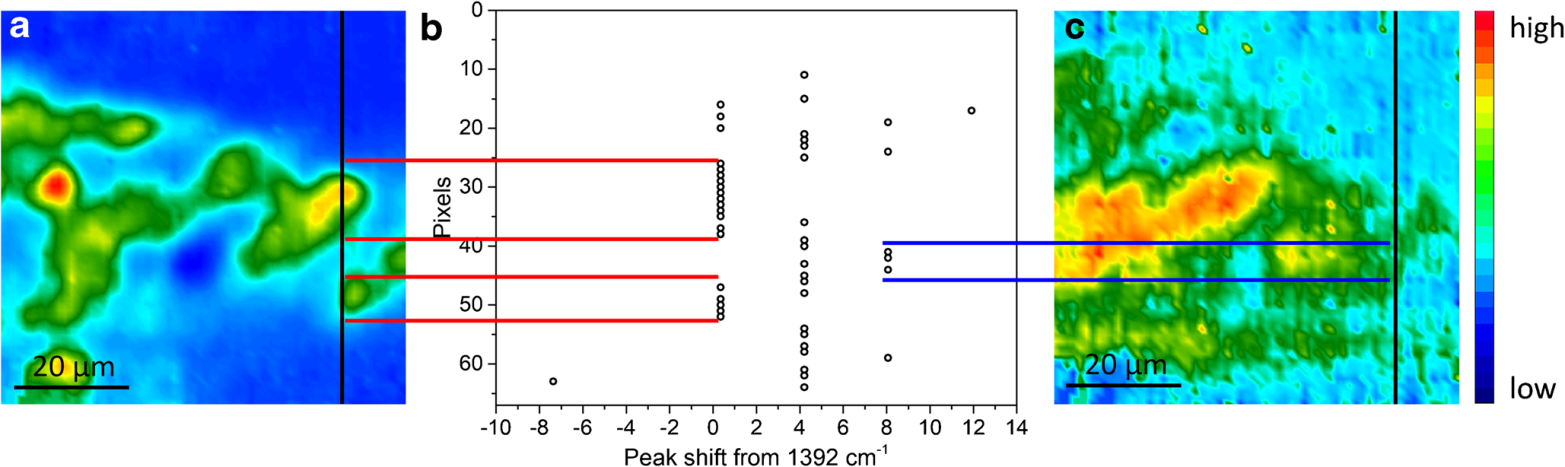
Peak shift of the spectral band corresponding to lead white depending on the formation of lead carboxylates. (**a**) Chemical image showing distribution of lead white pigment (integrated band: 1480–1200 cm^−1^). (**b**) Graph showing the peak shift of lead white. (**c**) Chemical image showing distribution of lead carboxylates (integrated band: 1560–1480 cm^−1^). The two vertical black lines in (**a**) and (**c**) indicate the ATR-FTIR spectra investigated to create graph (**b**). The maximum absorbance of the *ν*_as_(CO_3_^2−^) band centred at 1392 cm^−1^ corresponds to the area where lead white is clearly present (red horizontal lines), while in the regions where lead carboxylates are more dominant (blue horizontal lines), lead white is still present but with its *ν*_as_(CO_3_^2−^) peak shifted to higher wavenumbers

Figure 4e is obtained by evaluating the peak shift of the *ν*_as_(CO_3_^2−^) band with respect to the aforementioned reference band at 1384 cm^−1^ along an horizontal (black) line in the same area of measurement, as shown in Fig. 4d and f. It can be seen that there is no peak shift for spectra from areas of high absorbance of intact lead white; while in regions where lead white gradually decays to lead soaps, the *ν*_as_(CO_3_^2−^) spectral peak shifts by 4 cm^−1^ or 8 cm^−1^ to the higher wavenumber regions, showing the clear formation of a shell/ring of decayed lead white all around the core of the intact lead white pigment. This peak shift can be attributed to the variation of crystalline lead white to a more disordered structure at the interface, due to the extraction of metal ions from the pigment carried out by carboxylate groups of the ionomer-like binder and by the free saturated fatty acids (SFAs) in the binder. The presence of free SFAs is also demonstrated by the detection of a shoulder peak at 1707 cm^−1^ in the ATR-FTIR spectra of the oil binder (Fig. 4c). This band is ascribed to the presence of free fatty acids (*ν* C=O of COOH) naturally present in siccative oils (such as palmitic or stearic acids), or formed through oxidation and hydrolysis of the ionomer-like binder (azelaic acid) [1]. In addition, in regions mainly occupied by the oil binder and lead soaps, peaks of lead white are still present, even though at a significantly lower absorbance. In these regions, a substantial peak shift of lead white is observed, reaching up to + 19 cm^−1^ or + 23 cm^−1^ from the reference peak at 1384 cm^−1^. This remarkable peak shift can be correlated to an advanced decay in the binder regions, where micrometric (~ 1–5 μm) and sub-micrometric particles of lead white are dispersed in mixture with bigger grains (~ 50–100 μm; cfr SEM-BES image of Fig. 4b), and, on the basis of their reduced size and high reaction specific surface, resulting in them experiencing a higher rate of metal ion extraction. It is conceivable that the progressive peak shift of the *ν*_as_(CO_3_^2−^) spectral band from the core to the interface might occur not only for big grains of pigment but also in the case of large agglomerates of smaller particles of lead white. In fact, the phenomenon of peak shift can be observed in paint layers where the lead white particles show a very fine and well-sorted grain size (~ 10 μm), as in the case of layer 5 of sample A (Fig. 5). Here, the *ν*_as_(CO_3_^2−^) of lead white is centred at 1392 cm^−1^ and ATR-FTIR spectra collected in the core of the pigment always show vibrational bands due to metal soaps, indicating that, at this point, all fine-grained particles are partially decayed. Notwithstanding of the more advanced decay of the white pigment and of its fine grain size, the peak shift of lead white reaches + 8 cm^−1^ in regions characterized by a high presence of lead soaps, hence demonstrating the high chemical specificity of micro-ATR-FTIR spectroscopic imaging to study slight chemical differences even down to the microscale and on complex mixtures.

**Fig. 6:**
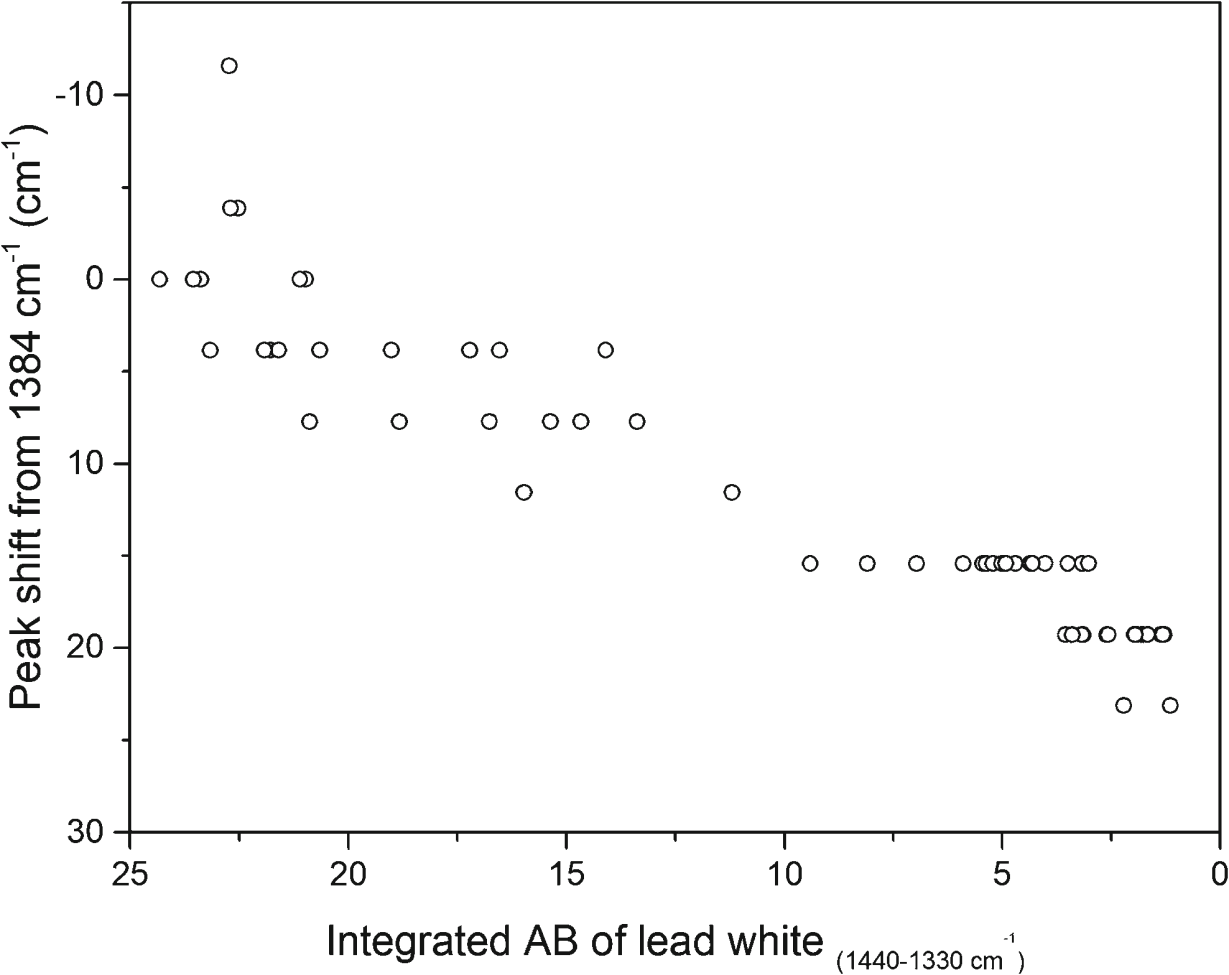
Graph showing the integrated absorbance of the *ν*_as_(CO_3_^2−^) of lead white in the range 1330–1440 cm^−1^ versus the peak shift observed in the core of intact lead white. Each point in the graphs corresponds to a single pixel of the FPA image. The ATR-FTIR spectra used for these graphs are extracted from the line of the FPA image drawn as a black horizontal line in Fig. 4d, f

As the extent of peak shift of the *ν*_as_(CO_3_^2−^) band is suspected to be correlated with a reduction in its quantity, the peak shift of the *ν*_as_(CO_3_^2−^) observed in the core of intact lead white against its integrated absorbance is plotted in Fig. 6, by sorting the values of integrated absorbance in decreasing order along the horizontal (black) line in Fig. 4d, f. As expected, the peak is at the reference (1384 cm^−1^) when the highest values of integrated absorbance for the *ν*_as_(CO_3_^2−^) are recorded, corresponding to the core of the intact lead white. When the integrated absorbance decreases, e.g. between 20 and 13, a peak shift was seen, e.g., of + 4 cm^−1^ and + 8 cm^−1^. Figure 6 supports our speculation that the peak shift of the *ν*_as_(CO_3_^2−^) is indeed directly dependent on the amount present in the painted layer. In short, while the chemical images are useful in providing the spatial information, quantitative information can be gathered from the ATR-FTIR spectra to give a detailed picture of the heterogeneity and composition of the sample.

## Conclusions

In this study, the possible contribution of moisture to the formation of lead soaps in complex stratigraphies containing multiple lead-based paint layers is suggested.

Thanks to the high-quality datasets supplied by micro-ATR-FTIR spectroscopic imaging and the particular experimental configuration used in this research, it was possible to discriminate and to localize the saponification products based on the different lead white pigments and to retrace the particular conservation history of these artefacts. Furthermore, as the samples shared similar composition/exposure to the same environmental conditions and in the light of other researchers work, it is hypothesised that the saponification of selected layers is linked to past restorations, to the complex stratigraphic succession (consisting of mixtures of inorganic and organic compounds), and to the presence of hydrophilic layers within the stratigraphy itself.

Finally, by exploring changes to the features of the target bands in the ATR-FTIR spectra, the decay suffered by the lead white pigments was assessed on the basis of their grain size, the presence of the contiguous layers, and the source of humidity. The analytical advancements provided by the spectroscopic approach here proposed increase the amount and quality of information that can be extracted from FTIR imaging datasets. Furthermore, this new spectroscopic approach could be easily applied to different systems, permitting the evaluation of compositional modifications of other compounds (other pigments, binders, varnishes), even in complex matrices.

Moving from the analytical challenges faced in this study to a wider scenario, the results generated are of high interdisciplinary impact. In fact, by allowing a holistic approach, they shed new light on manufacturing processes, interaction with environmental conditions, restoration events, and choices of vocational iconography made by the painters and conservators.

## Supplementary Information

ESM 1 (PDF 267 kb).
