# Supplementary material for: Insight into the effects of moisture and layer build-up on the formation of lead soaps using micro-ATR-FTIR spectroscopic imaging of complex painted stratigraphies
Source: Anal Bioanal Chem. 2020 Nov 9;413(2):455–67. doi: 10.1007/s00216-020-03016-6 (PMC7806535; doi:10.1007/s00216-020-03016-6)
Supplement: Supplementary file 1 — (PDF 267 kb). [file 216_2020_3016_MOESM1_ESM.pdf]

DOI: 10.1007/s00216-020-03016-6

Supplementary materials: Analytical and Bioanalytical Chemistry

Insight into the effects of moisture and layer build-up on the formation of lead soaps using micro ATR-FTIR spectroscopic imaging of complex painted stratigraphies

Elena Possenti[1], Chiara Colombo[1], Marco Realini[1], Cai Li Song[2] and Sergei G. Kazarian[2]

[1] *Istituto di Scienze del Patrimonio Culturale, Consiglio Nazionale delle Ricerche, ISPC-CNR, Via R. Cozzi 53 Milan, 20125, Italy*

[2] *Imperial College London, Department of Chemical Engineering, South Kensington Campus London SW7 2AZ, United Kingdom*

corresponding authors: elena.possenti@cnr.it

s.kazarian@imperial.ac.uk

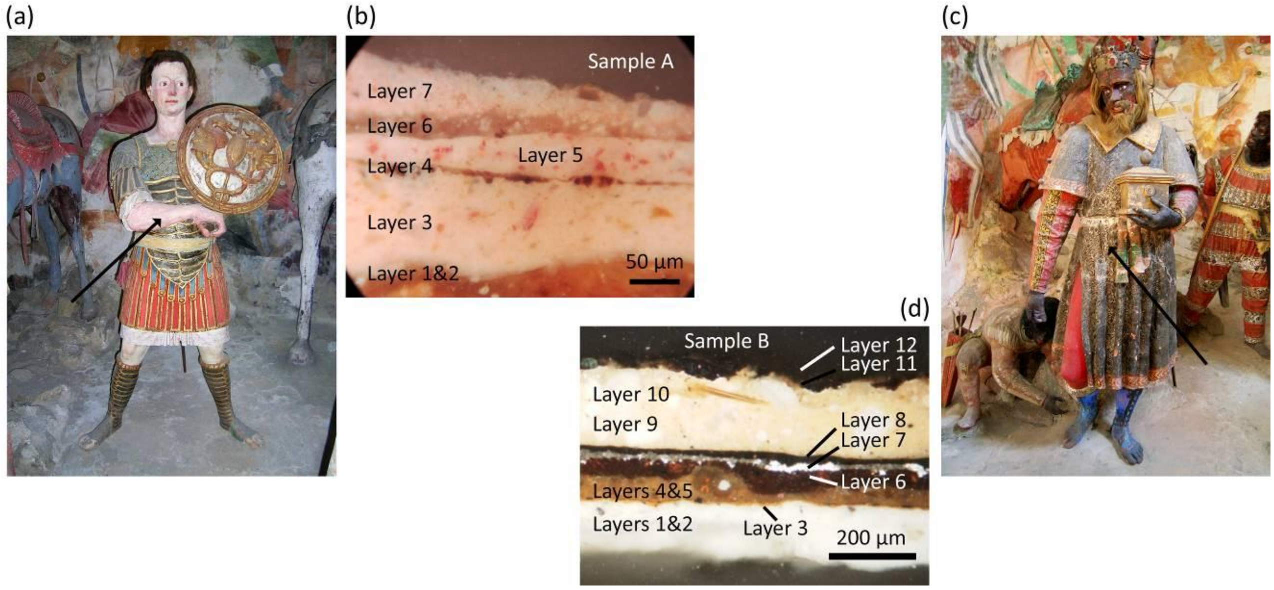

Fig. S1 Sacred mount of Varallo, Chapel V:(a) statue of the soldier and (b) polished cross section of sample A; (c) statue of the wise king and (d) polished cross section of sample B. The black arrows in (a) and (c) indicate the sampling regions of the two samples

DOI: 10.1007/s00216-020-03016-6

Table S1 Sample names and pigment composition of the paint layers

Sample name	Layer number	Colour	Pigment composition	Results from micro-ATR-FTIR spectroscopic imaging
A	Substrate: terracotta	Brown	/	
	1 (inner)	Light brown	*Organic*	
	2	Light brown	*Organic*	
	3	Light pink	Lead-based white pigment, gypsum, red ochre, cinnabar	Basic lead carbonate, lead carbonate, siccative oil, lead soaps
	4	Brownish	*Organic*	Protein
	5	Light pink	Lead-based white pigment, gypsum, red ochre, cinnabar, minium	Basic lead carbonate, siccative oil, lead soaps
	6	White-grey	Gypsum	Gypsum, calcium oxalates, protein
	7 (outer)	Light pink	Lead-based white pigment, gypsum, red ochre	Basic lead carbonate, siccative oil, lead soaps
B	Substrate: terracotta	Brown	/	
	1 (inner)	White	Lead-based white pigment, silicates	
	2	White	Lead-based white pigment, silicates	
	3	Light orange	Lead-based white pigment, red ochre	
	4	Brownish	*Organic*	
	5	Light orange	Minium, red ochre	
	6	Brownish	*Organic*	
	7	Metal leaf	Tin	
	8	Brownish	*Organic*	
	9	White-yellow	Lead-based white pigment, red ochre	
	10	White-yellow	Lead-based white pigment, red ochre	Basic lead carbonate, siccative oil, lead soaps
	11	Metal leaf	Silver	
	12 (outer)	Dark	Deposit	

DOI: 10.1007/s00216-020-03016-6

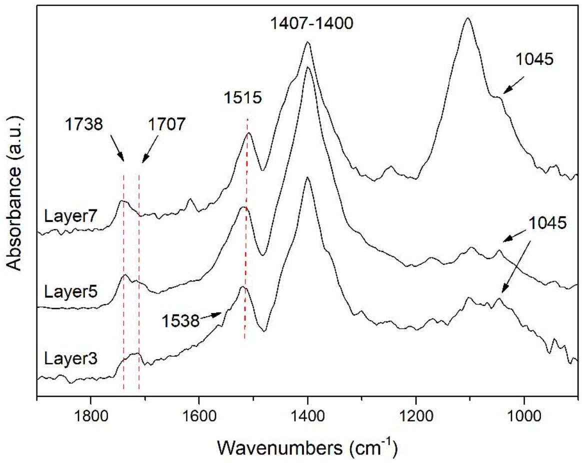

Fig. S2 Sample A. Normalized ATR-FTIR spectra showing the characteristic $\nu_{as}(COO^{-})$ band of lead soaps at 1515 cm^{-1} in a mixture with oil and lead white detected in layers 3, 5 and 7
